# Social Media Guidelines for Anatomists

**DOI:** 10.1002/ase.1948

**Published:** 2020-05-06

**Authors:** Catherine M. Hennessy, Danielle F. Royer, Amanda J. Meyer, Claire F. Smith

**Affiliations:** ^1^ Department of Anatomy Brighton and Sussex Medical School University of Sussex Brighton United Kingdom; ^2^ Department of Cell and Developmental Biology University of Colorado School of Medicine Aurora Colorado; ^3^ School of Human Sciences The University of Western Australia Perth Australia

**Keywords:** Social media, Gross anatomy education, Anatomical sciences education, Medical education, Professionalism, Medical ethics, Informed consent, Cadavers

## Abstract

Social Media has changed the way that individuals interact with each other ‐ it has brought considerable benefits, yet also some challenges. Social media in anatomy has enabled anatomists all over the world to engage, interact and form new collaborations that otherwise would not have been possible. In a relatively small discipline where individuals may be working as the only anatomist in an institution, having such a virtual community can be important. Social media is also being used as a means for anatomists to communicate with the current generation of students as well as members of the public. Posting appropriate content is one of the challenges raised by social media use in anatomy. Human cadaveric material is frequently shared on social media and there is divided opinion among anatomists on whether or not such content is appropriate. This article explores the uses and challenges of social media use in the field of anatomy and outlines guidelines on how social media can be used by anatomists globally, while maintaining professional and ethical standards. Creating global guidelines has shown to be difficult due to the differences in international law for the use of human tissue and also the irregularities in acquiring informed consent for capturing and sharing cadaveric images. These nuances may explain why cadaveric images are frequently shared on social media. This article proposes that as standard practice, anatomists obtain informed consent from donors before sharing images of cadaveric material on social media and ensure posts include a statement stating the same.

## INTRODUCTION

Over the past 15 years, the rise of social media has radically changed how students and educators source, disseminate and communicate information with each other over. Social media are internet‐based tools, such as websites and applications that allow users to retrieve, explore, and actively participate in content creation, editing and dissemination (including information, ideas, personal messages, images, and videos), through open and often real‐time collaboration with other users (McGee and Begg, 2008; Ventola, 2014). Social media include (but are not limited to) platforms such as Facebook (Facebook, Inc., Menlo Park, CA), WeChat (Tencent Holding Ltd., Shenzhen, China), Twitter (Twitter Inc., San Francisco, CA), Reddit (Advanced Publications, San Francisco, CA), Instagram (Facebook, Inc., Menlo Park, CA), Snapchat (Snap Inc., Santa Monica, CA), YouTube (Google LLC, San Bruno, CA), LINE Messenger (Line Corp., Tokyo, Japan), and WhatsApp (Facebook Inc., Menlo Park, CA). With the ever‐increasing accessibility of the Internet, the number of social media users worldwide has been steadily increasing. In 2010, there were 0.97 billion users, in 2018 there were 2.62 billion users and it is estimated that there will be over three billion users by 2021, equaling over one‐third of the global population (Wagner, 2018). The reasons to use social media vary but some of the most popular reasons are surrounding social media enabling three distinct but interacting functions to occur instantaneously: sourcing of information, dissemination of information, and communication (Statista, 2019). When considering these within higher education they raise a number of challenges for educators to consider including a lack of control for students sourcing correct and relevant content versus fake news or inaccurate information (Allcott et al., 2019). Social media use has also been identified as being associated with an increase in mental health issues such as anxiety and depression (Dhir et al., 2018) and in education, this may result in students and educators alike comparing themselves to their peers and feeling dissatisfied and unhappy. Despite these challenges, social media offer many benefits to educators and are increasingly being used for communicating with students and networking with colleagues alike.

### Higher Education

The majority of students in education today have grown up with social media, including the liberal information sharing and rapid means of communication it offers (Cunningham and Shirley, 2015). Numerous investigations have shown that the majority of students in higher education use social media to acquire and share information and to communicate with peers (Jaffar, 2012; Hall et al., 2013; Foley et al., 2014; Mukhopadhyay et al., 2014; Barry et al., 2016). Across higher education, social media are being used by educators as educational tools to meet the learning needs of today's students (Junco et al., 2011; Forkosh‐Baruch and Hershkovitz, 2012; Arquero and Romero‐Frias, 2013; Sarapin and Morris, 2015; Keenan et al., 2018). For educators, social media allows “one‐to‐many” dissemination to hundreds of students in one post encouraging students to engage in course content in a way that other tools cannot (McArthur and Bostedo‐Conway, 2012).

### Medical Education

Within medical education, large majorities of medical students have consistently reported using social media platforms for learning (Bosslet et al., 2011; George et al., 2013), with Facebook, YouTube, and Twitter being reported as the most commonly used by medical students (El Bialy and Jalali, 2015; Al Wahab et al., 2016), although Instagram and Snapchat are also used (Knight‐McCord et al., 2016). Medical educators have increasingly been incorporating the use of social media into their teaching practice and in a similar trend to student use, Facebook, YouTube, and Twitter have shown to be the most commonly used by educators (Curran et al., 2017; Sutherland and Jalali, 2017). Consequently, there has been an increase in research articles since 2010 exploring the challenges and benefits of using social media in medical education (Cheston et al., 2013; Hollinderbaumer et al., 2013; Madanick, 2015; Roy et al., 2016; Curran et al., 2017; Sutherland and Jalali, 2017).

The mutual outcomes of these review articles are that social media are positively received by students and the following benefits are commonly reported: fostered active collaborative learning through engagement in user‐generated content; enhanced student engagement, communication and feedback opportunities; and increased access to resources without location restrictions. Similar benefits were reported by El Bialy and Jalali (2015) however they point to a “disconnect” between educators and students use of social media with educators mostly focusing on posting videos, articles and explanatory comments on their educational social media platforms, whereas students place more value in the social media posts from educators containing quizzes and revision files. Arnbjörnsson (2014) reported that there is no consistent evidence that social media interventions increase assessment scores and questioned why, despite this, social media interventions are so favorably taken up by students. This may be explained by Kaczmarczyk et al. (2013) suggestion, that students' attitudes and behaviors surrounding information sharing and communication using social media are likely to be reflected in their approach toward academic tasks.

Sterling et al. (2017) conducted a review of social media use in graduate medical education and identified the following benefits: education and learning (specifically promoting clinical concepts and technical skills); disseminating evidenced‐based information; and circulating conference material and supporting journal clubs. Sterling's review also highlighted that social media are used as a screening tool for recruitment into residency programs and issues around professionalism, highlighting that as students' progress through their medical education social media behavior begins to have an influence on the careers of medical students. Additionally, a review by Roy et al. (2016) reported that the most prevalent challenge was the potential for social media to adversely affect medical professionalism. However, since medical students and young doctors are avid social media users, it has been argued that this is further reason for medical educators to begin modeling social media professionalism or “digital professionalism” (Ellaway et al., 2015) through the use of social media in educational settings (MacDonald et al., 2010; Walji and Stanbrook, 2015).

### Anatomy Education

Social media are becoming increasingly established in the field of anatomy as educational aids to anatomy educators (Chytas, 2019). Facebook pages created by anatomists have shown to be popular with medical students, with the majority of the student users perceiving that interacting with the anatomy education pages helped their learning (Jaffar, 2014; Pickering and Bickerdike, 2017). The majority of interactions on Facebook pages were students asking questions about anatomy (Pickering and Bickerdike, 2017). A neuroanatomy course‐specific Twitter hashtag set up by Hennessy et al. (2016) was found to increase student engagement with the course content and boost morale by acting as a supportive network for students. This was an unanticipated but important finding for Hennessy et al. (2016) who were conscious that the notoriously difficult content a neuroanatomy course delivers has been shown to induce “neurophobia” (Javaid et al., 2017). Similar to the findings of Kind et al. (2014), students also valued how Twitter facilitated quick and easy communication between educators and students (Hennessy et al., 2016).

A common trend observed across several studies is that the majority of students merely observe educational Facebook and Twitter platforms and the small proportion of students who engage more with the platforms, generating more “likes,” comments and discussions tend to be the high‐achieving students (Michikyan et al., 2015; Hennessy et al., 2016; Jaffar and Eladl, 2016; Pickering and Bickerdike, 2017). Hennessy et al. (2016) suggested that disengagement with social media platforms could be used as a way to identify underperforming students early in the medical education. However, there appears to be a natural decline in the uptake of such designated educational platforms by more recent student cohorts, categorized as Generation Z (Iqbal, 2018). Border et al. (2019) suggested various reasons for the decline including “social media fatigue” (Bright et al., 2015), which has been attributed to students being bombarded with online educational resources and feeling a need to use all of them due to a “fear of missing out” (Bright et al., 2015). It is not surprising then that students can feel overwhelmed by the vast amount of online education resources available to them and become more selective and conservative with their social media use (Border et al., 2019). Instagram has been identified as the favored social media platform for the current generation of students and it has the potential for offering the same learning support opportunities as Facebook, Twitter, and YouTube, however, as yet there has been no student evaluation data published on the educational value of Instagram for anatomy education (Douglas et al., 2019).

### Accuracy of Anatomy Information on Social Media

YouTube is a social media platform which continues to be widely used by medical students to source anatomy educational videos and students have reported valuing these readily available resources for helping their understanding of anatomy (Jaffar, 2012; Barry et al., 2016). However, anatomists have raised concerns regarding the accuracy and educational quality of the anatomy videos available on YouTube (Azer, 2012; Raikos and Waidyasekara, 2014). Similar concerns have been raised for the quality of anatomy information available on Wikipedia, one of the highest used social media sites for online learning (Choi‐Lundberg et al., 2016), with only one‐third of anatomy articles being classified as “good” and many others containing inaccurate or missing information (Suwannakhan et al., 2020). Chytas (2019) concluded that in order for social media to benefit students' anatomy learning effectively, guidance should be provided by educators to ensure that the material being taught is of appropriate quality. The availability and immediacy of educators have also been emphasized for the successful use of educational social media platforms (McArthur and Bostedo‐Conway, 2012; Hennessy et al., 2016).

### Social Media Use by Anatomy Educators and Associations

Increasing numbers of anatomists and clinicians involved in anatomy teaching are engaging in social media for professional networking and to build a community of practice (Keenan et al., 2018; Marsland and Lazarus, 2018). It has been recognized by medical associations that social media provides opportunities to connect with members, disseminates information, and promotes recent research more widely, due to the international reach of social media (Carroll et al., 2016; Sutherland and Jalali, 2017). Scientific journals including *Anatomical Sciences Education* now have active social media (predominantly Twitter) accounts (@AnatSciEduc), and educators frequently tweet about the publication of a recent article including the journal's Twitter handle for increased exposure. Similarly, anatomical associations worldwide have social media accounts and there has been a noticeable rise in the use of conference‐specific hashtags by anatomical associations at their respective meetings. Many associations encourage conference delegates to live‐tweet using the conference hashtag to allow further information dissemination and networking opportunities for members (Jalali and Wood, 2013; Jalali et al., 2015). Table 1 summarizes some of the international anatomy association social media accounts and conference hashtags.

**Table 1:** Twitter Accounts and Meeting Hashtags Being Used by International Anatomy Associations

Association Name	Twitter Handle	Meeting Hashtag Template
		(Example)
American Association of Clinical Anatomists (AACA)	@AACAnatomy	#ClinAnatYR
		(e.g., #ClinAnat19)
Anatomical Society (AS)	@anat_soc	#AnatSocSEASONYR
		(e.g., #AnatSocWinter18)
Australian and New Zealand Association of Clinical Anatomists (ANZACA)	@ANZACA_Inc	#ANZACAYEAR
		(e.g., #ANZACA2018)
American Association for Anatomy (AAA)	@AnatomyOrg	#anatomyYR
		(e.g., #anatomy17)
British Association of Clinical Anatomists (BACA)	@BACA_Anatomy	#BACAYEAR
		(e.g., #BACA2018)
International Federation for the Association of Anatomists (IFAA)	@IFAA2019	#IFAAYEAR
	@ifaa2021	(e.g., #IFAA2019)

Benjamin and Royer (2018) described how The American Association of Clinical Anatomists (AACA) Twitter account (@AACAnatomy) has successfully employed daily tweets and conference hashtags to build a larger network of professionals (including anatomists, health care providers, and scientists), increase the accessibility of anatomical research and education within both professional and lay communities, and better engage with the association's membership. The Anatomical Society (Great Britain and Ireland) reported increases in the number of users and contributors to their conference Twitter hashtags (Keenan et al., 2017) and concluded that although Twitter is not widely used to initiate dialogue, it is being used to network and share research ideas and plays a key role in the modernization of academic organizations. The International Federation of Anatomy Associations (IFAA) and Federative International Program for Anatomical Education (FIPAE) recently launched the Global Anatomy Learning Excellence Network (GALEN) Twitter account (@GALENnetwork) as a platform to share and communicate any content related to anatomy education. Although there is a lack of literature reporting the impact of such social media accounts and hashtags, anatomical associations are increasingly recognizing the benefits of social media including: facilitating global communication and networking and providing a medium for anatomists to communicate, share and receive up‐to‐date information on anatomy research quickly and concisely with international colleagues (IFAA, 2019). Effectively, social media are facilitating the creation of a global anatomy community.

### Images of Human Cadavers on Social Media

As with all digital technologies, the benefits of social media use in anatomy are accompanied by challenges. One of the emerging issues in the field of anatomy is that images of human cadaveric material or cadavers are being shared on public, anatomy‐related, social media accounts (Bond, 2013; Anonymous, 2014; Hutchinson, 2018). This has clear ethical implications for the anatomy profession since there is no explanation of where the cadavers were sourced or whether consent was received from donors to share such images (Hildebrandt, 2019). For clarity, within this article cadaveric material or cadavers are considered to be any human material from the deceased (embalmed or fresh frozen, whole body or isolated parts), which has been accepted by health science institutions for education and research.

Hildebrandt (2019) has compared sharing cadaveric material on social media to the continued use of unclaimed bodies (Bernstein, 2016a,b) and the for‐profit or “body brokers” at work in the United States (US) (Champney, 2019) and has termed these “abusive practices” because there is a common lack of informed consent gathered from the deceased individuals regarding how anatomists use the bodies in question. However, some anatomy educators have argued that sharing cadaveric dissections on social media maximizes the wishes of donors (that their bodies are used for education) due to the fact that a greater potential audience can be reached, and believe that this, in turn, negates any ethical concerns regarding sharing cadaveric dissections on social media (Rai et al., 2019).

### Purpose of Human Cadavers on Social Media

**Figure 1:**
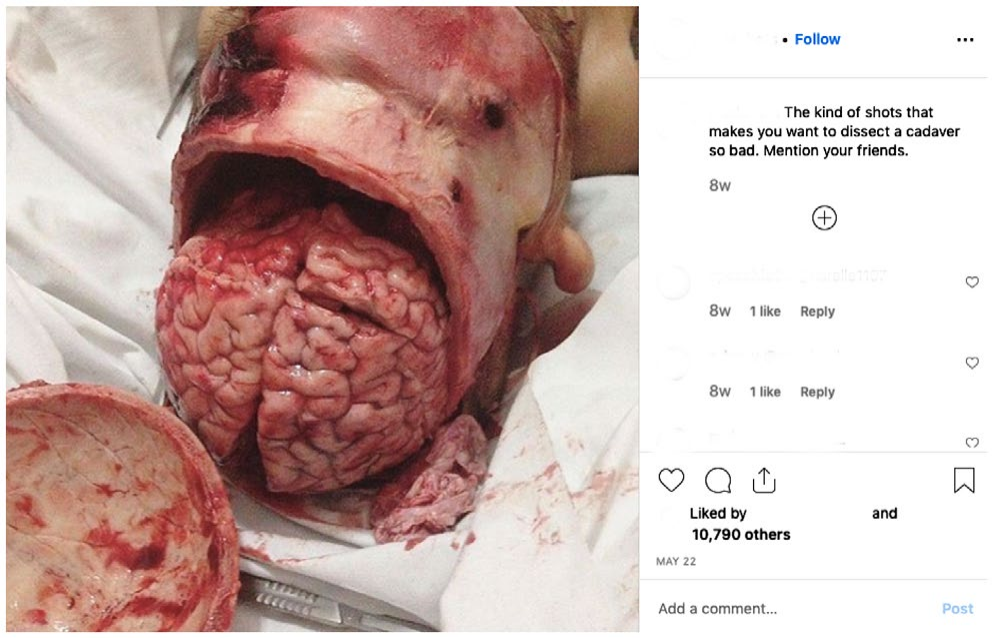
Image with little educational content posted publicly on the @Medshots Instagram account.

**Figure 2:**
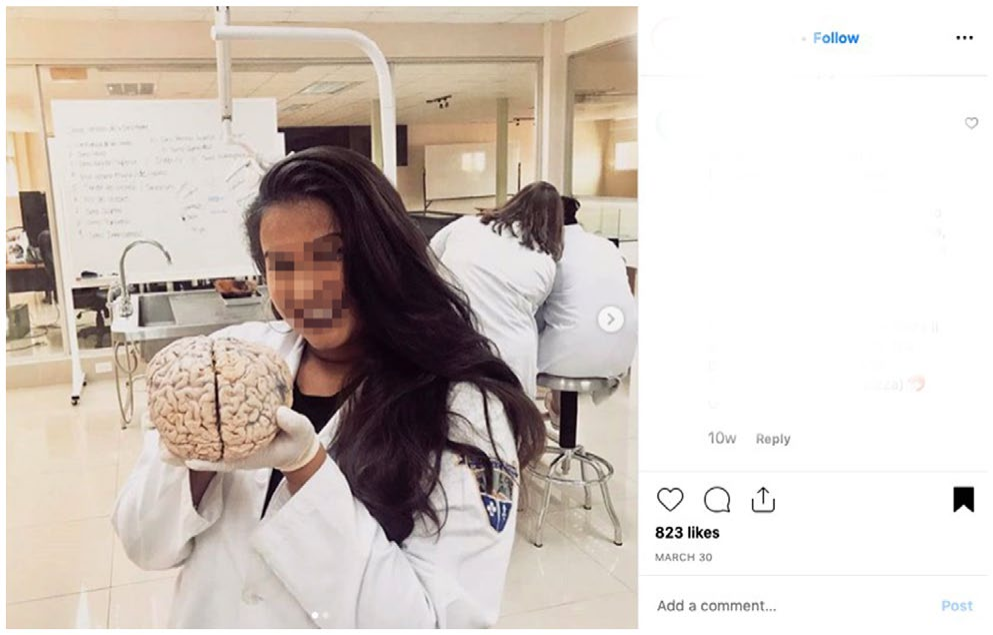
Image of a student holding a human brain posted publicly on the #medlife Instagram account.

**Figure 3:**
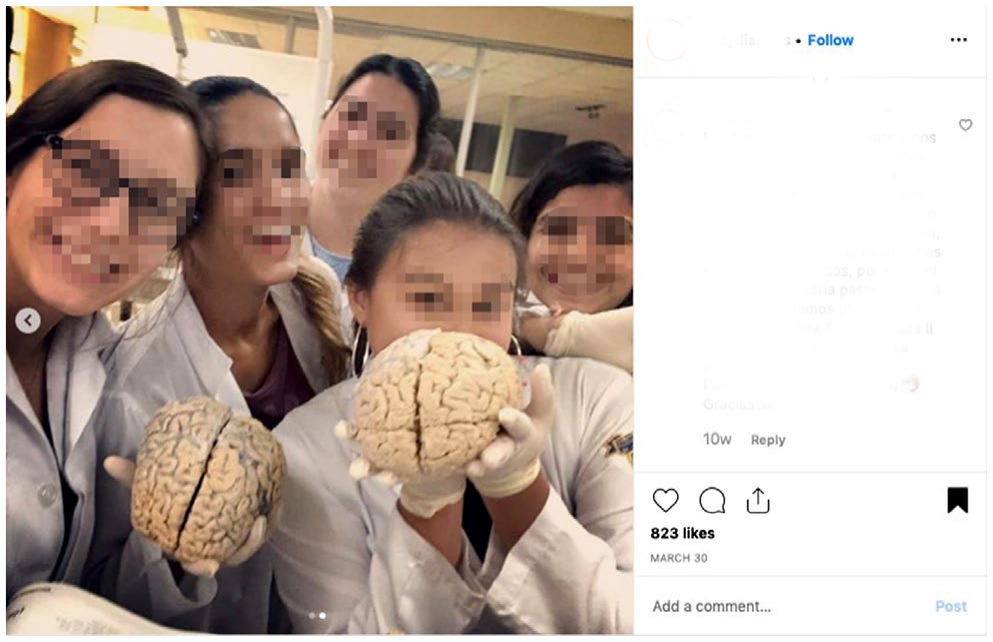
Image of a group of students holding human brains posted publicly on the #medlife Instagram account.

In our experience as anatomy educators and social media users, it is common to see cadaveric images on social media which do not provide educational content and therefore do not support the intentions of the act of donation. For example, some Instagram accounts (@medshots) and hashtags (#medlife) commonly share images which have little educational value and are perhaps more gratuitous in nature (see Figs. 1, 2, 3). Cadaveric images can be quite explicit, particularly if seen by members of the public who are not accustomed to observing human cadaveric material, and images such as that demonstrated in Figures 1, 2, 3 have the potential to negatively impact the public's perception of the level of respect anatomists have for donors (Hildebrandt, 2019).

**Figure 4:**
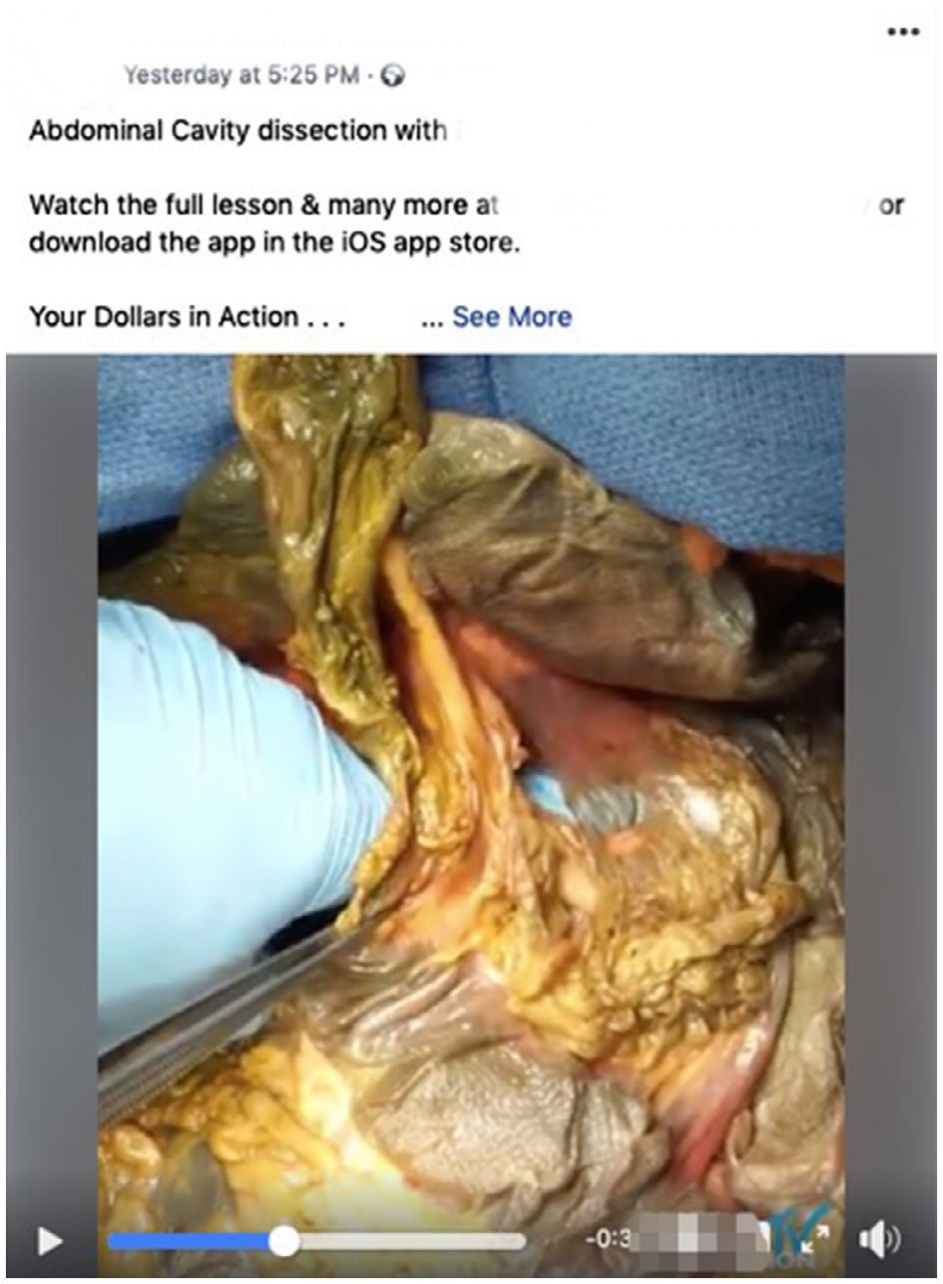
Stilled image of one of the dissection videos publicly available on the Seattle Science Foundation Facebook page. This video demonstrates and explains the epiploic foramen connecting the greater and lesser sacs of the abdominal cavity.

Some social media accounts such as the Seattle Science Foundation Facebook page share cadaveric dissections publicly specifically for educational purposes (see Fig. 4). Rai et al. (2019) surveyed 300 members of the public who follow the Seattle Science Foundation's Facebook page and reported that 98% agreed that cadaveric material including dissection should be accessible by the public for anatomy education. However, regarding the respondents of this survey, 18% were healthcare providers and 76% were students (Rai et al., 2019). A majority of respondents (85%) stated that such content was not too graphic for untrained eyes, however, it is unclear from their results where healthcare providers and students stood on this matter (Rai et al., 2019). It is plausible that the healthcare providers with more experience and awareness of codes of conduct were the 18% of respondents who felt the use of cadaveric specimens was too graphic for Facebook and Instagram (Rai et al., 2019).

### Privacy and Confidentiality

In medicine, a similar debate is ongoing in the field of pathology where it has been argued that the societal and professional benefits of sharing pathological images of human patients, e.g., diseased lungs on cigarette packets, overwhelmingly outweighs the potential for harm when patient privacy is maintained by making the image unidentifiable (Crane and Gardner, 2016). Like anatomists, pathologists experience difficulties in receiving informed consent from patients since pathologists do not always have direct contact with patients. Crane and Gardner (2016) believe that having policies in place which demand that patient consent is obtained before posting patient images on social media would severely restrict pathology education. However, the repercussions of sharing images of patients without consent were highlighted in a recent case where a man discovered an image of his amputated leg was being used as a health warning on a cigarette packets in France (BBC, 2019). Although the intention, in this case, was to promote health, the man and his family who were able to identify the image due to characteristic scars, felt “betrayed” and “stunned”. In a similar way, an anatomist may believe that it is ethical to share human cadaveric material if the content being shared is for educational purposes, however without informed consent the professionalism and ethical standards of the profession come into question. Another challenge that anatomy educators face when posting cadaveric material on social media is the risk of breaching confidentiality laws. National and local laws regarding maintaining confidentiality exist such as the Health Insurance Portability and Accountability Act (HIPPA) of 1996 in the United States (USDHHS, 2003) and General Data Protection Regulation (GDPR) of 2018 in the United Kingdom (UK) and Europe (ICO, 2018), to ensure that personal information or data belonging to any individual are processed and stored lawfully and in accordance with the reason why the information was obtained. The same lack of clarity exists on whether donor information, including cadaveric images, was obtained to be shared on social media.

As the challenges of social media use in anatomy have emerged, so too have guidelines on social media use by individual anatomy associations for their respective members. For example, the AACA have pinned a link to their guidelines on the @AACAnatomy Twitter account (AACA, 2016) and the AS, IFAA (incorporating GALEN) and the American Association for Anatomy (AAA) have published guidelines on their websites (AAA, 2019; IFAA, 2019; Hennessy et al., 2019a). Associations do not want to be linked or hold affiliations with any social media use which could be deemed unprofessional, unethical or bring the profession into disrepute. Recently, Cornwall and Hildebrandt (2019) highlighted the need for continued discussion around the ethical challenges arising from digital technologies infiltrating anatomy education. With social media showing no signs of diminishing, there has been a call for guidelines on how anatomists internationally can use social media effectively, while maintaining professional and ethical standards. Despite the publication of guidelines by individual anatomy associations, images of human cadavers continue to be published on social media and one of the aims of this article is to explore why this is by reviewing the guidelines from international anatomy associations and identifying where any nuances lie. This article also aims to propose global guidelines for anatomists on social media use to maintain ethical and professional standards of the anatomy profession.

## MATERIALS AND METHODS

### Review of the Guidelines from International Anatomy Associations

A web search for the social media guidelines provided by anatomy associations listed on the International Federation for the Association of Anatomists (IFAA) website (societies pages) was conducted. The web search was confined to English‐speaking associations which included: The Anatomical Society (AS), The Anatomical Society of South Africa (ASSA), The American Association for Anatomy (AAA), The American Association of Clinical Anatomists (AACA), The Australian and New Zealand Association of Clinical Anatomists (ANZACA), The British Association of Clinical Anatomists (BACA) and The IFAA itself. Thematic analysis was conducted on the relevant guidance documents found which involved: reading each document three times, coding keywords or phrases, categorizing codes into subtheme and condensing subthemes into broader common themes (Braun and Clarke, 2006).

### Review of the Guidelines from International Medical Governing Bodies

**Figure 5:**
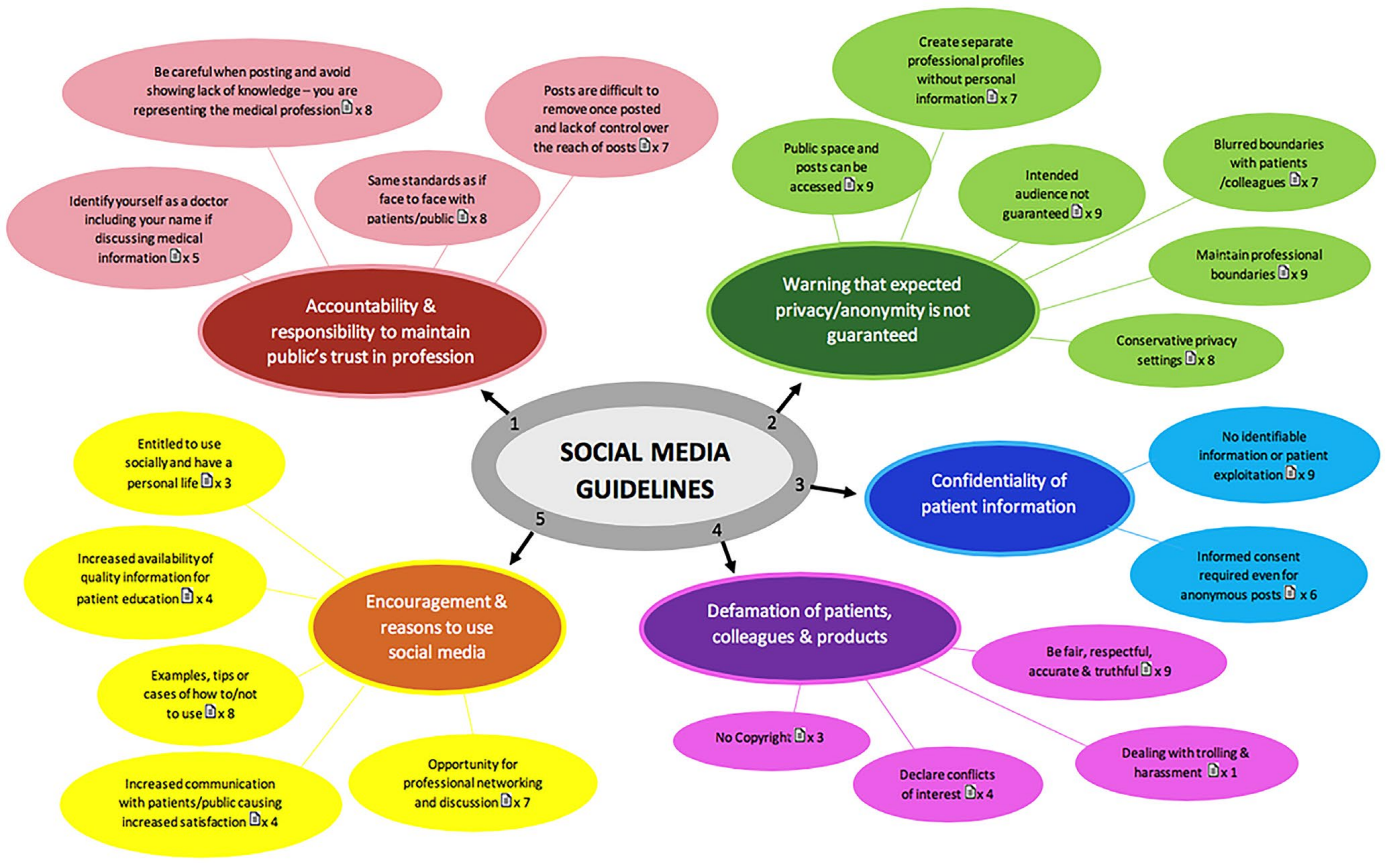
Themes and subthemes identified in medical guidance documents on social media use. The main themes featured in each of the documents, unlike the subthemes. The document symbol and number indicate the number of documents which featured each subtheme (Hennessy et al., 2019b).

Also, due to the close relationship anatomists have with the medical profession (regarding the responsibility of respecting confidentially and ensuring anonymity when sharing patient information), guidelines from English‐speaking international medical governing bodies were also used to inform this article. The authors (C.M.H. and C.F.S.) have previously published a review of the guidance documents provided by the main medical governing bodies in English‐speaking countries, which identified nine guidance documents as detailed in Table 2 (Hennessy et al., 2019b). A summary of the main themes and subthemes of the medical guidance documents is illustrated in Figure 5 (Hennessy et al., 2019b).

**Table 2:** International Medical Governing Bodies and Their Reviewed Guidance Documents

Governing Body	Document Title
General Medical Council (UK)	Doctor's Use of Social Media (GMC, 2013).
British Medical Association	Social media: Practical Guidance and Best Practice (BMA, 2018a). Social Media, Ethics and Professionalism (BMA, 2018b).
Canadian Medical Association	Social Media and Canadian Physicians: Issues and Rules of Engagement (CMA, 2011).
Canadian Federation of Medical Students	CFMS Guide to Medial Professionalism: Recommendations for Social Media (Brasg, 2013).
Australian Medical Association and New Zealand Medical Association	Social Media and the Medical Profession (Mansfield et al., 2011).
American College of Physicians	Online Medical Professionalism: Patient and Public Relationships: Policy Statement from the American College of Physicians and the Federation of State Medical Boards (Farnan et al., 2013).
American Medical Association	Professionalism Guidelines for Social Media Use: A Starting Point (Kind, 2015).
Federation of State Medical Boards (US)	Model Guidelines for the Appropriate Use of Social Media and Social Networking in Medical Practice (FSMB, 2012).

## RESULTS

Four guidance documents were identified from anatomy associations as detailed in Table 3. No guidance documents were found published by The Anatomical Society of South Africa (ASSA), The Australian and New Zealand Association of Clinical Anatomists (NZACA) and The British Association of Clinical Anatomists (BACA). The following seven common themes and subthemes were identified from the guidance documents:

1. Social media use is encouraged since it is advantageous for promoting anatomical science and facilitating communication within the anatomy community.
2. A respectful environment where no harm is caused to colleagues should be maintained.
3. Confidential information which threatens privacy of colleagues should not be shared.
4. Informed consent must be received before any confidential information or intellectual property owned by colleagues is posted on social media.
5. Professional standards should be maintained in order to avoid harming public perception of the anatomy profession. Social media posts containing personal or political views and items that are off‐topic should be limited.
6. Social media posts should be well considered prior to posting due to the vast reach and permanency of social media content.
7. Social media posts containing cadaveric material should be sensitively handled and deeply considered before being posted on social media.

**Table 3:** The Social Media Guidance Documents Published by International Anatomy Associations Which Were Reviewed and Analyzed for Common Themes

Association	Document title
American Association of Clinical Anatomists (AACA)	AACA Twitter Guidelines for Engagement of The Membership and Public (AACA, 2016).
Anatomical Society (AS)	Social Media Guidelines for Engagement with Membership and Members of the Public (Hennessy et al., 2019a).
American Association for Anatomy (AAA)	American Association of Anatomists. Social Media Use (AAA, 2019).
International Federation for the Association of Anatomists (IFAA)	Social Media Guidelines for the IFAA: Engagement with Members and the Public (IFAA, 2019).

The first six themes listed above featured in all the guidelines from medical governing bodies. Any such common key messages from both the anatomy associations and medical governing bodies were used to develop the suggested global guidelines, which are listed in the Appendix of this article.

### Key Differences

The medical guidelines have a strong focus on protecting patients. For example, the guideline regarding confidential information and maintaining privacy, the medical governing bodies are mostly concerned about maintaining the privacy of patients and not leaking confidential information, whereas the anatomy associations focus on maintaining the privacy of colleagues and not leaking any personal information and intellectual property owned by other colleagues. Similarly, the guideline regarding informed consent focusing on receiving consent from patients before posting any patient information whereas for the anatomy governing bodies the informed consent guideline focuses on receiving consent from colleagues before posting any information or photos about colleagues including research outputs.

The medical guidelines also contain several additional recommendations regarding upholding the public confidence and trust in the profession such as “avoid showing a lack of knowledge”. The medical guidelines also recommend adopting conservative privacy settings and suggest creating separate personal and professional accounts to maintain professional boundaries with patients and the public.

One unique theme in the anatomy guidelines pertains to the handling of cadaveric content. There are conflicting messages in the guidelines from anatomy associations on whether posting cadaveric material on social media is appropriate and acceptable or not. The AACA guidelines warn members that such content can be expected to be seen on their social media platforms but does not give any recommendations to others on posting and sharing cadaveric content. The AAA guidelines do not cover cadaveric content however posting cadaveric content is not listed as one of the items considered inappropriate to post. The Anatomical Society (AS) states clearly that posts containing cadaveric material are prohibited by their members and the Society states that they do not want any association with such posts due to uncertainty around informed consent being received from the donor. The IFAA, being an international body, state that content containing cadaveric material should be handled sensitively and highlight that since laws and body donation programs vary greatly internationally, anatomists must make a professional judgment based on their local laws and their own ethical beliefs when deciding on posting cadaveric material.

These conflicts made the creation of global guidelines for how social media should be used in the field of anatomy a challenging task and highlight that more discussion needs to happen around reaching a consensus for how anatomists handle cadaveric material on social media.

## DISCUSSION

The motivation for this article was to create universal guidelines on social media use for all anatomists globally. In reviewing the guidelines produced for the wider medical field, one difference that became apparent between the medical sector and anatomy is that medical professionals (on the whole) work with live patients, who typically have the ability to grant consent for medical professionals to share anonymized patient information on social media. In contrast, anatomists typically work with human cadavers, that is, deceased individuals who can no longer give consent. The unique access anatomists have to cadavers brings additional ethical considerations for how social media can be used by anatomists, particularly regarding posts containing cadaveric material. There is a level of privilege granted to anyone who views human cadaveric material and this privilege is lost when images or videos of cadavers are shared on public social media sites.

The existing guidelines contain conflicting recommendations on whether posts containing cadaveric material are appropriate to post on social media which might be explained by the differing laws worldwide surrounding obtaining informed consent from donors for capturing and publicizing images. To demonstrate this a comparison between laws in the United Kingdom (UK), United States (US) and Australia will now be discussed.

### Anatomy Law

#### United Kingdom and Europe

In the UK, the Human Tissue Authority (HTA) regulates all anatomy laboratories and their use of cadavers. The HTA website clearly specifies that donors should expect that their bodies will be used for anatomical examinations to teach healthcare students and professionals, scientific research and surgical training of healthcare professionals (HTA, 2019a). In the UK, donors have the opportunity to give consent on donation forms (which vary across institutions) for images to be taken and used in education and research. The London Anatomy Office (LAO) donation form allows donors to tick a box to give consent for images to be taken with the understanding that donors will not be identifiable in the images (LAO, 2015). However, the HTA requires specific written consent from donors for their body or body parts to be displayed in public, for example in a licensed gallery or museum premises, to provide assurance to the public that such specimens are handled with care and treated with respect (HTA, 2019b). This means that donation forms from any UK institutions who hold a license for the public display of cadavers must include a section for donors to grant consent for public display.

#### United States

In the US the Uniform Anatomical Gift Act (UAGA) 2006, created in 1968 and revised most recently in 2009, provides federal‐level regulatory guidelines for body donations for educational, medical, and scientific endeavors (UAGA, 2006). However, each state may decline or modify the act, and each state independently enforces and regulates any licensing bodies associated with body donation. Currently, the majority of states have adopted the 2006 UAGA with modifications, leading to substantial national variation in regulations pertaining to organ, tissue and body donation (ULC, 2019). For example, some states such as Colorado are significantly less regulated in this arena (e.g., does not require licensure for funeral directors), while other states are more heavily regulated. The UAGA specifies that body donations may be made to an accredited school, hospital, organ procurement organization or non‐profit for the purpose of research or education. The Act does not propose a standard donation form, or provide direct guidance on donor photography, the public dissemination of such materials, or the need for informed consent beyond the general purpose of research and education. At the institutional level, willed body programs in the US typically require their donor recipients to adhere to more specific policies, which may include limits on images and their dissemination. The Colorado State Anatomical Board (SAB), housed at the University of Colorado School of Medicine where one of the coauthors is based (D.F.R.), does not currently provide the option for donors to consent to image capture or sharing directly. Rather, consent is deemed granted as part of the general body donation for “use at the discretion of the Board for education, research, or other scientific purposes.” However, the SAB Cadaver Agreement requires recipients to receive SAB approval for the capture and dissemination of donor images, and places the following restrictions on donor photography: no student photography, intended for educational purposes only (broadly defined), no identifying features including the face, should be cropped as close as possible for the structure or region of interest. Dissection videos must adhere to the above requirements, and also be hosted on a secure server with access controlled by an institution for a specific‐targeted audience.

#### Australia

Like in the United States, each of Australia's eight states and territories are governed by their own laws on body donation and the use of human tissue, none of which provide guidance on taking and sharing cadaveric images. An up‐to‐date National Anatomy Act would be welcomed by anatomists in Australia but it will be a long time coming, if ever, with this matter being of low priority to politicians and law‐makers. Therefore, it is up to anatomists and institutions to instill policies regarding the ethical use and imaging of cadavers. One of the co‐authors (A.J.M.) is based at The University of Western Australia (UWA) which operates under the Anatomy Act of Western Australia written in 1930. The University of Western Australia has generated their own Body Donation Consent Forms and rules of conduct in the human anatomy laboratories. The university's donation program website states that bequests will be used to educate specific medical and healthcare students and professionals but does not explicitly cover image capture or use (UWA, 2019). At UWA, like many other anatomy laboratories, cameras or devices that could capture images are banned from the anatomy laboratory to prevent images of body donors being obtained and distributed without their consent. A ban on image‐capturing devices in human anatomy laboratories seems appropriate since donations forms at UWA, like in Colorado, do not ask donors for consent to capture and disseminate images.

Another consideration highlighted in a study by Habicht et al. (2018) is that body donation is the exclusive source of cadavers in anatomy departments in only 32% of countries worldwide, and in 57% of countries (including the US) unclaimed bodies are part of or the exclusive source of cadavers. Where body donation programs are in place, donors may have had the opportunity to sign consent for images to be captured and used for educational purposes, however as just discussed, this is not guaranteed depending on the country. Given the variation in the levels of informed consent received from donors and the use of unclaimed bodies worldwide, it is highly unlikely that informed consent for capturing images containing cadaveric material (even if for the advancement of anatomy education and research), and publicizing them on social media has been received from the deceased individuals.

### Donor Expectations

Donors are likely to have donated their bodies with certain expectations of how their body will be used. The HTA website clearly states that only certain students and groups of healthcare professionals can have access to cadaveric material which is likely to set the expectations of UK donors (HTA, 2019a). Donor information packs (LAO, 2015) are also frequently used to set expectations and inform UK donors about how their bodies will be used and by whom, including imaging and public display. However, to our knowledge, information packs rarely include a statement informing UK donors that images may be shared on social media. As already discussed, donors in the US and Australia are likely to have unclear expectations, if any, regarding how images of their body are used and shared, including on social media. In Colorado, the SAB deems the broad consent for educational use that a donor sign to include the capture and sharing of images, as long as the intent is education.

This raises questions regarding transparency for donors and has implications on the legality of sharing human cadaveric material on social media. Information and images shared on social media are largely regarded as being in the public domain. Importantly, the aforementioned anatomical social media accounts which share cadaveric material are public accounts, meaning any member of the public who is a social media user can access and view such content. Furthermore, social media has no geographical borders which means that the laws and cultures of sharing cadaveric content are not contained within countries or states. Two of the authors are anatomists working in the UK where they believe the culture of sharing cadaveric images on social media is rarely acceptable and likewise the guidance from The Anatomical Society (of Britain and Ireland) prohibits members from sharing cadaveric material (Hennessy et al., 2019a). However, UK‐based anatomists are likely to regularly come across cadaveric material on social media perhaps because it was considered acceptable in the country where the post originated. However, the argument remains that a donor, regardless of their country of origin, may not have anticipated that images of their donated body would be shared so publicly and globally on social media. Jones (2019) raised the same argument about donor expectations in reference to using donor bodies for creating 3D printed anatomy models stating that donors are likely to expect their bodies will be used for local medical and healthcare education rather than prints or images of their body being spread and sold worldwide. Cornwall et al. (2016) have also argued that anatomists are at risk of giving an impression to the public that the value of body donation is undermined by anatomists using donors so indiscriminately. Jones (2019) has suggested that explicit informed consent must be received from donors ahead of creating anatomical 3D printed material and the distribution of anatomical 3D prints “should be accompanied by a statement regarding details of the consent provided by body donors and an acknowledgment of the body donor's contribution” to anatomy education, a suggested standard practice which is transferrable to posting cadaveric material on social media.

Posts containing human cadaveric images including live dissections are regularly circulated on social media and to the authors' knowledge, there is rarely an accompanying statement declaring that informed consent had been obtained from the donor. It is unclear why this is: perhaps laziness on behalf of the publisher; perhaps a lack of an efficient way to include a statement of informed consent due to the character limit of a platform like Twitter or perhaps consent is not always deemed a requirement. The latter seems the mostly likely due to the variance in regulations internationally surrounding obtaining informed consent for capturing and publicizing images of cadaveric material. Having a mindset that informed consent for such images is not a requirement must change due to the ethical and professional implications for the anatomy profession (Cornwall et al., 2016; Jones, 2019). The authors urge anatomists to ask for informed consent to be made explicit when they observe cadaveric images or videos on social media without an adjoining consent statement and that such a statement be included if anatomists are sharing cadaveric material themselves.

### Maintaining Ethical Standards

It must not be forgotten that for many years anatomy had a dubious public image due to the illegal procurement of bodies for anatomical examination (Persaud, 1984, 1997), which reinstates the importance and need for anatomists to explain where bodies have come from (Hildebrandt, 2019) and what is done to bodies once donated to maintain ethical standards (Barilan, 2005). The introduction of informed consent has allowed the profession of anatomy to become more transparent, reputable and one which recognizes that anatomical cadaveric materials are only available due to the altruism of donors and their families. However, there have been several recent reports questioning the ethical standards in anatomy, not only due to the increased use of digital technologies such as 3D printing and social media being used to share donor information without consent (Jones, 2019) but also due to the emergence of “body brokers” in the US who solicit bodies from hospices, hospitals, and nursing homes and sell them for profit to anatomy departments (Champney, 2019). Champney (2019) highlighted the need for heightened awareness around respect for bodies and has proposed that anatomists practice a standardized “bioethos” worldwide where donors are treated with respect and dignity rather than as material objects. Although “body brokers” claim that their motive is to improve healthcare education, the ethical ethos of the business has been criticized over recent years due to reports of abuse toward bodies, the large profits earned (Grow and Shiffman, 2017; Shiffman and Levinson, 2018) and the fact the individuals concerned have not altruistically donated their bodies (Champney, 2019). The IFAA have also recommended that only donated bodies should be used for anatomy teaching and research worldwide (IFAA, 2012).

In support of the bioethos described by Champney (2019), Jones (2019) added that respecting the wishes of the donor and their families is paramount in maintaining ethical standards and that anatomists must not give the impression to the public that the profession is losing sight of the gift that donors give to the anatomical profession. Accordingly, anatomists must ask donors and their families what their wishes are regarding posting images on social media to facilitate transparency with donors and the public about how cadaveric material is going to be used.

Arguably, cadaveric images published in an anatomy atlases are comparable to sharing cadaveric images on social media for educational purposes, and there is rarely (if ever) information on the origin of cadavers (Jones et al., 2019) let alone a statement indicating that informed consent has been received from donors for publication (e.g., Moore et al., 2014; Gilroy and MacPherson, 2016). This may be linked to how anatomists coped to distance themselves when working with cadavers historically. Dating back to the eighteenth century the Scottish anatomist William Hunter first described developing the need for a “certain inhumanity” toward cadavers as a way of coping with the act of dissection (Richardson, 2000). This same strategy of learning anatomy by disengaging with or detaching humanistic inquiry toward cadavers became a standard practice among anatomists and medical trainees for many years in the United States (Hafferty, 1988; Walter, 2004; Štrkalj, 2016). However, such strategies are no longer encouraged and on the contrary, anatomy education is increasingly recognized as having a key role in helping medical students learn about medical ethics (Cornwall and Hildebrandt, 2019), where students confront issues such as death and dying, empathy, respect, and dignity for donors (Hildebrandt, 2019). Removing the humanity connected to donors, therefore, does not work in modern anatomy education and by the same token anatomy, educators should strive to ensure that if cadaveric images are shared on social media, that the deceased individual gets recognition and was fully informed and agreeable for their body to be used in this way (Hildebrandt, 2019).

### Limitation of the Study

Social media use within the profession of anatomy is an emerging topic and as a result this study could only source four published guidance documents on social media use from anatomy associations. This may have limited the worldwide perspective on how social media should be used in anatomy. The authors have drawn from their research and experience of using social media as anatomy educators in three separate countries, however, we acknowledge that anatomy educators in other countries may view social media sharing of cadaveric images differently. As more anatomy associations worldwide publish guidelines on social media use, the global guidelines suggested in this study should also be reviewed. Also, due to the rapid evolution of social media, global guidelines on social media use for anatomists must be continuously reviewed and updated.

## CONCLUSIONS

As anatomists, we must be mindful that we depend on body donations from the public and we must maintain a level of trust from the public regarding how we treat human cadaveric material. Sharing cadaveric material which is clearly non‐educational on social media is arguably unethical. Furthermore, it is highly unlikely that informed consent to share images on social media has been received from the donors or unclaimed bodies. Social media is contributing to the globalization of anatomy. Additionally, the public nature of social media means that the profession of anatomy is being forced to be more transparent with donors regarding the potential of capturing and sharing of cadaveric material on social media.

Producing guidelines that are suitable for global use by anatomists is extremely challenging due to varying laws and cultures between and within countries. Being compliant with the human tissue laws from the country in which you work is likely to be best practice, however, we recommend that cadaveric images and videos should only be shared on social media if informed consent from donors has been received. We propose that this should become standard practice if anatomists are sharing cadaveric material on social media and that posts should be accompanied with a statement declaring informed consent had been received by the donor. Furthermore, we recommend that anatomists ask for informed consent to be made explicit when they observe cadaveric images or videos on social media without an adjoining statement that informed consent had been received.

Below are the guidelines recommended for the proactive, safe, and ethical use of social media by anatomists worldwide, to avoid reducing the levels of professionalism and public's trust in the anatomy profession.

## NOTES ON CONTRIBUTORS

CATHERINE M. HENNESSY, B.Sc. (Hons), M.Sc., P.C.A.P., F.H.E.A., is a lecturer of anatomy at Brighton and Sussex Medical School in Brighton, UK. She teaches anatomy to undergraduate and postgraduate entry medical and allied health science students. She is conducting a part‐time PhD exploring how medical students and doctors develop their understanding of social media professionalism and the development of a professional online identity. Her other research interests include developing anatomy core curricula for health professionals.

DANIELLE F. ROYER, B.Sc. (Hons), M.A., Ph.D., is an associate professor in the Department of Cell and Developmental Biology at the University of Colorado School of Medicine. She directs the medical anatomy block for first‐year medical students and teaches gross anatomy and ultrasound to graduate students in the Modern Human Anatomy Master of Science program. Her research is focused on ultrasound, anatomical variation, and teaching practices in clinical anatomy and anatomy education.

AMANDA J. MEYER, B.Sc. (Hons), Ph.D., M.R.S.B., is a lecturer in the School of Human Sciences at The University of Western Australia, Perth, Western Australia, Australia. Previously, she was a lecturer in the School of Health Professions at Murdoch University, Perth, Western Australia, Australia for six years. She teaches gross anatomy, histology, and neuroanatomy. Her research interests are in assessment, teaching, and student perceptions and motivations.

CLAIRE F. SMITH B.Sc., P.G.C.E., Ph.D., S.F.H.E.A., F.A.S., F.L.F., N.T.F., is a professor and Head of Anatomy at Brighton and Sussex Medical School (Brighton), Falmer, United Kingdom. She is a fellow of the Anatomical Society and a member of the Court of Examiners for the Royal College of Surgeons England. She is Secretary General for the European Federation of Experimental Morphologists and the lead author on Gray's Surface Anatomy and Ultrasound textbook. Her research is in understanding the learning experience.

## APPENDIX A

### Anatomy Social Media Guidelines

#### General

Each individual user is accountable for their own posts and the impression their posts give of themselves and any professional bodies they are associated with. One common rule of thumb used by professionals on social media is “if I wouldn't tell it to my mother or my boss or publish it in the newspaper, then I wouldn't post it on social media.” It is always worth bearing in mind that many social media platforms are within the public domain and therefore consideration must be given to the potential vast reach of posts. Furthermore, posts are generally permanent, even if deleted by users, because social media platforms store posts indefinitely, meaning that posts can always be tracked and traced. Therefore, “think before you post”, since posts can potentially be viewed by anyone for any length of time and can be captured by screenshot by another social media user before deleted.

#### Profile

Where possible you should state on your social media profiles that “views are my own” (or to that effect), so as to not be deemed to be supported or associated with any other professional body (e.g., your employer or associated university or anatomical association).

#### Networking

Social media is a useful way for anatomists to share opinions, teaching resources, research outputs, and career achievements. This form of online networking may lead to successful international work collaborations.

#### Copyright of Research Data

Photos, videos, or other media for which the individual is not the copyright holder should not be shared without permission from the copyright holder. This includes research data presented at conference presentations. Anatomists should make it explicit during conference presentations if images are not allowed to be captured and shared. Reposting copyright information which has already been posted by the owner is acceptable.

#### Defamation

Avoid posting defaming, offensive, vulgar, harassing, or threatening language, personal attacks or accusations, or derogatory terms targeting individuals or groups, in the field of anatomy or otherwise.

#### Conflicts of Interest

Any conflicts of interest must be declared if promoting anatomy products or resources on social media.

#### Confidentiality and Privacy

Confidential information that threatens an individual's privacy should never be posted (e.g., home address, credit card numbers). Informed consent should be received from any individuals referred to in posts through text or images.

#### Cadaveric Images

Photos and videos of cadavers or cadaveric material must be sensitively handled. Legislation on public display of cadaveric material varies internationally and therefore you may come across social media posts containing images of cadaveric material. While such content has the potential to educate the public about anatomy and body donation, it can also adversely affect the relationship between the public and body donation programs. It is, therefore, a professional judgment based on the laws and guidance under which individuals work and on ethical considerations as to whether it is appropriate to post, “like” or “repost” such posts. However, cadaveric images and videos should only be shared on social media if informed consent from donors has been received and this should be explicitly stated on an adjoining statement.

Some social media platforms have the option to label media as “sensitive content,” which provides a warning to viewers and affords them the opportunity to better control what they see. Such options should be considered when sharing media of cadaveric material on public sites, even when the intent is educational. Remember, while you may be accustomed to seeing cadaveric material, the general public is not, and may consider such content to be offensive.
